# Noninvasive High-Throughput Single-Cell Analysis of HIV Protease Activity Using Ratiometric Flow Cytometry

**DOI:** 10.3390/s131216330

**Published:** 2013-11-28

**Authors:** Rok Gaber, Andreja Majerle, Roman Jerala, Mojca Benčina

**Affiliations:** 1 Laboratory of Biotechnology, National Institute of Chemistry, Ljubljana 1000, Slovenia; E-Mails: rok.gaber@ki.si (R.G.); andreja.majerle@ki.si (A.M.); roman.jerala@ki.si (R.J.); 2 Center of Excellence EN-FIST, Ljubljana 1000, Slovenia

**Keywords:** mCerulean-mCitrine FRET-HIV protease sensor, ratiometric flow cytometry, sensitize emission FRET

## Abstract

To effectively fight against the human immunodeficiency virus infection/acquired immunodeficiency syndrome (HIV/AIDS) epidemic, ongoing development of novel HIV protease inhibitors is required. Inexpensive high-throughput screening assays are needed to quickly scan large sets of chemicals for potential inhibitors. We have developed a Förster resonance energy transfer (FRET)-based, HIV protease-sensitive sensor using a combination of a fluorescent protein pair, namely mCerulean and mCitrine. Through extensive *in vitro* characterization, we show that the FRET-HIV sensor can be used in HIV protease screening assays. Furthermore, we have used the FRET-HIV sensor for intracellular quantitative detection of HIV protease activity in living cells, which more closely resembles an actual viral infection than an *in vitro* assay. We have developed a high-throughput method that employs a ratiometric flow cytometry for analyzing large populations of cells that express the FRET-HIV sensor. The method enables FRET measurement of single cells with high sensitivity and speed and should be used when subpopulation-specific intracellular activity of HIV protease needs to be estimated. In addition, we have used a confocal microscopy sensitized emission FRET technique to evaluate the usefulness of the FRET-HIV sensor for spatiotemporal detection of intracellular HIV protease activity.

## 1. Introduction

### 1.1. HIV Virus and HIV Protease

The human immunodeficiency virus (HIV), which causes the acquired immunodeficiency syndrome (AIDS) was first discovered in 1981. It is a huge health, economic, and social burden for many countries and communities worldwide. Today, HIV infection is treatable but not curable. Due to the rapid mutation rate of HIV, even highly active antiretroviral therapy fails in a significant number of patients [1]. HIV protease is crucial for the maturation of viral particles and therefore represents an important target for therapy. This protease recognizes specific peptide sequences between individual proteins in Gag and Gag-Pol polyproteins [2]. If the HIV protease is inhibited, viral particles still bud from the cellular membrane, but the proteins inside them are not in a mature and active form; therefore, the virus is not infective. Detailed studies of the HIV protease structure, maturation, and cleavage mechanism have resulted in the discovery of many HIV protease inhibitors, nine of which have been approved for clinical use [2]. The rapid mutation rate of the HIV virus results in great numbers of different viral variants in an individual patient. Unfortunately, resistance of the HIV protease to all registered protease inhibitors has already been reported; therefore, novel protease inhibitors must be developed in order to continue the fight against the HIV epidemic [2].

### 1.2. Methods for Measuring HIV Protease Activity and the Efficiency of Inhibitors

Potential HIV protease inhibitors are tested *in vitro* and *in vivo* before any type of clinical trial. There are direct and indirect *in vitro* methods to determine the efficiency of the inhibitors. Indirect methods are usually used to evaluate the ability of the substance to inhibit viral replication in the cell culture [3,4]. Several different primary human cell lines, such as the peripheral blood mononuclear, cord blood mononuclear, or MT-2 cells, are used in such assays [5,6]. The rate of viral replication is monitored by a viral p24 antigen capture assay or viral reverse transcriptase test or by observing cytotoxic effects on cell cultures caused by viral replication [3,6,7]. The main advantage of the cell line–based assays is that the experimental conditions are more realistic than in the *in vitro* assays that use recombinant HIV protease. However, these assays also have some significant disadvantages. Cell line-based assays are relatively expensive and laborious and are therefore not appropriate for massive screening experiments used for developing novel antiviral compounds. Moreover, indirect assays are used to evaluate the overall inhibitors ability to inhibit viral replication and not the specific protease inhibition characteristics of the tested compound.

Direct methods for measuring the HIV protease rely on synthetic peptides with a fluorescent molecule on one site and a quencher molecule on the other site of the HIV protease cleavage sequence. In synthetic sensors, 5-[(2-aminoethyl)amino]naphthalene-1-sulfonate (EDANS) and 4′-dimethyl- aminoazobenzene-4-carboxylate (DABCYL) are used as the fluorophore and quencher pair, respectively [8]. When linked together, DABCYL significantly reduces the fluorescence intensity of EDANS. When a synthetic polypeptide between EDANS and DABCYL is cleaved by the HIV protease, the fluorescence is recovered. Such synthetic substrates are relatively inexpensive and, in combination with a recombinant HIV protease, can be used in high-throughput assays for testing potential HIV protease inhibitors.

The HIV protease activity and efficiency of protease inhibitors can be analyzed *in vivo* using genetically encoded sensors. Two strategies have been developed. The first one is based on bioluminescence resonance energy transfer (BRET). In the BRET assay, humanized *Renilla reniformis* luciferase (hRLuc) is linked to humanized green fluorescent protein (hGFP2) with a polypeptide linker containing the HIV protease cleavage site [9]. After the addition of the hRLuc substrate, light emitted from hRLuc is transferred to hGFP2, which results in hGFP2 fluorescence. The intensity of hGFP2 fluorescence decreases when an active HIV protease cuts the polypeptide linker between hRLuc and hGFP2. Due to a low background, which is a general characteristic of luciferase-based assays, BRET-based HIV protease assay is very sensitive, but requires the addition of a synthetic hRLuc substrate. The second strategy for direct *in vivo* measuring of HIV protease activity is based on Förster resonance energy transfer (FRET). As in first strategy, a FRET sensor is constituted from two reporter fluorescent proteins covalently linked by a polypeptide linker containing an HIV protease cleavage site. The AcGFP1/mCherry pair [10] and AcGFP1/mCherry-mCherry triple combination [11] are used as FRET sensors for detecting HIV protease activity. The energy from the donor AcGFP1 protein is transferred to the acceptor mCherry, resulting in high fluorescence of mCherry even if the FRET protein pair is excited with light in the range of the excitation spectrum of AcGFP1. When the polypeptide link between the fluorescent proteins is cleaved, energy transfer is interrupted and fluorescence emission of the acceptor protein decreases. The advantage of FRET over BRET for *in vivo* detection of HIV protease activity is that no additional substrate is needed to measure the portion of the FRET sensor that is degraded; FRET is also applicable for spatial imaging in a single cell using microscopy.

The purpose of the current work is to develop rapid, high-throughput, noninvasive methods for measuring HIV protease activity and screening for protease inhibitors. In this paper, we describe methods for monitoring protease activity on the basis of a novel transgenic FRET-HIV protease-sensitive sensor based on a mCerulean and mCitrine FRET pair. The FRET efficiency of the sensor was analyzed using fluorescent spectroscopy, ratiometric flow cytometry, and confocal microscopy. We assessed the functionality of the FRET sensor in *in vitro* studies with a recombinant HIV protease and in *in vivo* studies of HIV protease activity within cells transfected with an HIV pseudoviral genome. In addition, we describe novel applications of the FRET-HIV sensor for studies of intracellular HIV protease activity. Using flow cytometry, we measured the percentage of the HIV pseudovirus-containing cells with intracellular HIV protease activity in a high-throughput manner. Such information could be important when studying the factors involved in the process of HIV dormancy inside the infected cells. In addition, the confocal microscopy sensitized emission FRET technique complemented the flow cytometry results, enabling imaging of protease activity within the cell. Our results demonstrate that the methods effectively detected and quantified the HIV protease activity of subpopulations and can therefore be used for scanning for potential inhibitors.

## 2. Experimental Section

### 2.1. Cloning of the FRET-HIV Protease Sensor and other Plasmids

**Figure 1.**
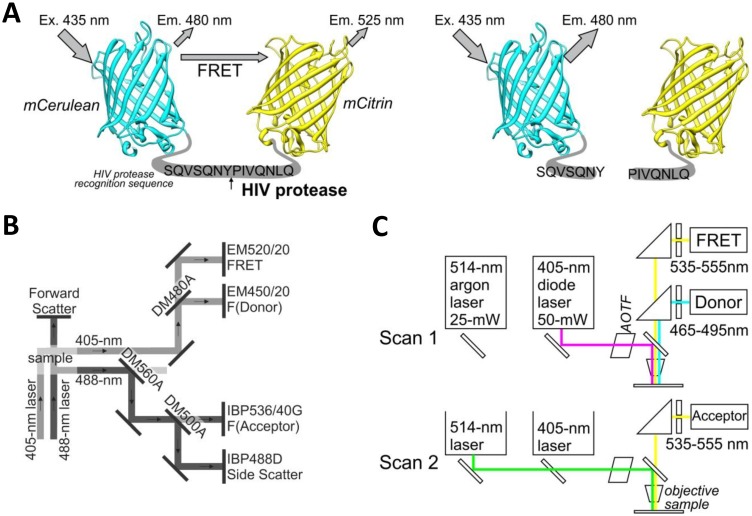
FRET-HIV protease-sensitive sensor. (**a**) The FRET-HIV sensor is composed of a donor mCerulean protein linked to an acceptor mCitrine with a peptide, which is a target site for the HIV protease. When excited with 433-nm light, the mCerulean emits light at 475 nm. In close proximity with the mCitrine, some energy is transferred to mCitrine, which then emits light at 529 nm. When the fusion protein is cleaved by the HIV protease, mCitrine is no longer in close proximity to mCerulean, resulting in a decrease of acceptor and a concomitant increase of donor fluorescence intensity. (**b**) Ratiometric flow cytometry setup with emission optics after excitation with 405-nm and 488-nm lasers with dichroic mirrors and filters. A dichroic mirror (DM480A) and a filter (EM520/20) for the emission light path at 405-nm excitation differed from the prefabricated version. And (**c**) Microscopic setup for imaging FRET-HIV sensor with emission optics after excitation with 405-nm and 514-nm lasers.

The yellow fluorescent protein mCitrine (mCit) was polymerase chain reaction (PCR) amplified from plasmid pRSET-B-mCitrine using forward primer (5′-CATGTCTAGAGCCGGCGTGAGCAAGGG CGAGGAGC) and reverse primer (5′-CATCTGCAGTTACTACTTGTACAGCTCGTCCATGCCG). The PCR product was cut with *Xba*I and *Pst*I restriction enzymes and ligated into equally cut plasmid BBa_I712015 (Registry of Standard Biological Parts) containing the HIV protease cleavage site with the amino acid sequence SQVSQNY↓PIVQNLQ. This sequence is also called the p17/p24 peptide [12], which is used in commercial synthetic HIV Protease Substrate 1 [sequence RE-(EDANS)- SQNY↓PIVQK-(DABCYL)-R] (H6660-1MG, Sigma, St. Louis, MO, USA), but it was not used in the FRET- or BRET-based sensors for intracellular detection of HIV protease activity [9–11]. This ligation resulted in a plasmid with mCitrine that proceeded with an in-frame HIV protease cleavage site (pHIV_CLS_-mCit). A cyan fluorescent protein, mCerulean (mCer), was PCR amplified from plasmid pEGFP-C1-mCerulean (Clontech Laboratories, Mountain View, CA, USA) using forward primer (5′-CATGGAATTCATGGTGAGCAAGGGCGAGGAGCTGTTCACC) and reverse primer (5′-CATGACTAGTCTTGTACAGCTCGTCCATGCCGAGAGTGATCC). The PCR product was cut using *Eco*RI and *Spe*I restriction enzymes and ligated into *Eco*RI and *Xba*I cut plasmid pHIV_CLS_-mCit to obtain an in-frame fusion of mCerulean-HIV_CLS_-mCitrine. The above-described process is a modified BioBrick cloning technique [13] that allows the creation of in-frame protein fusions. The FRET-HIV sensor was cut from the BioBrick vector using restriction enzymes *Eco*RI and *Spe*I and ligated into the mammalian expression vector pcDNA3, which was cut with *Eco*RI and *Xba*I, thereby generating pFRET-HIV (Figure 1A).

A plasmid pCeVe (plasmid backbone pRSET-B) [14] coding for mCerulean linked to yellow fluorescent protein mVenus with an 8-amino acid linker (MHGGSGGTE) that was not cleaved by the HIV protease was used as the FRET-control plasmid. The FRET-control was used as a negative control for the *in vitro* HIV protease assays. The pmCerulean and pmCitrine plasmids were used for donor- and acceptor-only controls, and for non-FRET control for the microscopy and flow cytometry. For *in vivo* expression of the HIV protease plasmid, pNL4-3.HSA.R^−^.E^−^ (pNL4-3) was used [15,16].

### 2.2. Cell Lines and Transfections

Human embryonic kidney cells (HEK) 293 and 293T were cultivated in DMEM (Invitrogen, Life technologies Corporation, Carlsbad, CA, USA) supplemented with 10% (v/v) fetal serum albumin (Invitrogen) at 37 °C in 5% CO_2_. The HEK293 cells were harvested from an actively growing culture and plated on 6-well tissue culture plates (TPP, Trasadingen, Switzerland) at 5 × 10^5^ cells per well or 8-well microscopic plates (ibidi Integrated BioDiagnostics, Martinsried, Germany) at 5 × 10^4^ cells per well. After 24 h at approximately 50% confluence, the cells were transfected with appropriate plasmid combinations using the jetPEI transfection reagent according to the manufacturer's instructions (Polyplus transfection, Illkirch-Graffenstaden, France).

For *in vivo* quantitative measurements, the HEK293 cells (seeded on 6-well plates) were transfected with pFRET-HIV (0.5 μg DNA/well) with pNL4-3 (1.0 μg DNA/well). For the control experiments, empty pcDNA3 plasmid was used instead of pNL4-3. Forty-eight hours after transfection, the cells were washed with the PBS buffer and collected and analyzed with a spectrofluorimeter.

For confocal microscopy, the HEK293T cells (seeded on 8-well microscopy chambers) were transfected with pFRET-HIV (22 or 35 ng DNA/well) without or with pNL4-3 (385 ng DNA/well). For controls, cells were transfected with plasmids coding for (i) donor only (40 ng mCerulean DNA/well); (ii) acceptor only (70 ng mCitrine DNA/well), and (iii) a non-FRET control (mixture of 40 ng mCerulean and 70 ng mCitrine DNA/well). A pcDNA3 was added to the transfection mixture to obtain at total of 407 ng DNA/well. Cells were analyzed 24 h after transfection.

For flow cytometry, the HEK293T cells were seeded on 6-well plates and transfected with pFRET-HIV (80 and 150 ng DNA/well) without and with pNL4-3 (0.8 and 1.5 μg DNA/well). For controls, cells were transfected with plasmids coding for (i) donor only (160 ng mCerulean DNA/well); (ii) acceptor only (240 ng mCitrine DNA/well); and (iii) a non-FRET control (160 ng mCerulean and 240 ng mCitrine DNA/well). A pcDNA3 was added to the transfection mixture to obtain a total of 1.9 μg DNA/well. The cells were washed and collected in the phosphate-buffered saline (PBS) buffer 24 h after transfection. Inhibitors dissolved in dimethyl sulfoxide (DMSO) were used at final concentrations of 1 μM or 5 μM. DMSO at a final concentration of 0.1% was added to the control cultures.

### 2.3. *FRET-HIV Protease-Sensitive Sensor and FRET Control for* in Vitro *Assays*

The FRET-HIV protease-sensitive sensor was isolated from the HEK293T cells transfected with pFRET-HIV. The HEK293T cells were seeded on a 10-mm mammalian cell culture plate (TPP), cultivated to 50% confluence, and transfected with pFRET-HIV. Three days after transfection, the medium was removed and the cells were resuspended in 5 mL of sterile water and subjected to three freeze-thaw cycles to lyse. After the lysis mixture was centrifuged for 10 min at 14,000 rpm, and the supernatant was stored at −80 °C.

The FRET-control was isolated from *Escherichia coli* BL21(DE3) pLysS cells, which were transformed with pCeVe. Transformants were cultivated in a 100-mL Luria Broth (LB) medium to optical density (OD) 0.7 at 37 °C and then induced with 1 mM isopropyl β-D-1-thiogalactopyranoside (IPTG) (the final concentration). After induction, the cells were cultivated for 18 h at 25 °C. Cells were then harvested and lysed using buffer containing 50 mM TRIS, 150 mM NaCl, 1% Triton X-100, lysozyme 1 mg/mL, and benzonase 25 U/mL (71205-3, Novagen, Darmstadt, Germany). Cells were incubated in 10 mL of a lysis buffer for 15 min at 25 °C. After incubation, the lysate was centrifuged for 10 min at 14,000 rpm. The supernatant was stored at −80 °C.

### 2.4. In Vitro *Detection of HIV Protease Activity and Activity of Its Inhibitors Using the FRET-HIV Sensor*

An *in vitro* characterization of the FRET-HIV sensor was conducted using a multiwell plate reader (SynergyMx, BioTek, Winooski, VT, USA). The fluorescent intensities of donor mCerulean and FRET (energy transfer to mCitrine) (to mVenus for FRET-control) were measured at 475 nm and 530 nm (a 9-nm band width), respectively, by exciting the sensor with a 433-nm light (a 9-nm band width). The FRET efficiency was defined as the ratio of the fluorescent intensity of the FRET/acceptor (530 nm) to that of the donor (475 nm) when excited with a 433-nm light.

The function of the FRET-HIV sensor *in vitro* was determined using HIV-1 recombinant protease (SRP2152-10UG, Sigma). Various concentrations of the FRET-HIV sensor (a stock concentration of 20 mg/mL) or the FRET-control sensor (a stock concentration of 36 mg/mL) were added to a reaction buffer with a total volume of 200 μL at a pH of 6.5. The FRET assay reaction buffer contained 150 mM sodium phosphate buffer with a pH of 6.5 (Na_2_HPO_4_/NaH_2_PO_4_ at molar ratio of 1:2.17), 1 mM of ethylenediaminetetraacetic acid (EDTA), 10% glycerol, 1 M of NaCl, and 1 mM of dithiothreitol (DTT). Digestion of the peptide cleavage site of the FRET-HIV sensor by the HIV protease was performed for 2 h at 37 °C. The kinetics of the reaction was followed for 3 h.

To assess whether the FRET-HIV sensor could ascertain the HIV-1 protease with inhibitors, ritonavir or saquinavir (dissolved in DMSO) (1 μM or 5 μM final concentrations) were added to the reaction mixture. The control reactions were also performed in the presence of 0.1% DMSO. The FRET signals generated from the sensor in the presence and absence of the inhibitors were measured and compared to determine whether the inhibitory effect of the HIV-1 protease by the inhibitors could be detected using the sensor.

HIV Protease Substrate 1 (H6660-1MG, Sigma) was used as an alternative method for *in vitro* detection of the HIV-1 protease activity. Measurements were performed according to manufacturer's instructions using various concentrations of recombinant HIV-1 protease.

### 2.5. Fluorescent Spectroscopy Used for in situ Quantitative Detection of the FRET-HIV Sensor Signal

For quantitative *in situ* measurement of HIV-1 protease activity, the HEK293 cells (seeded on a 6-well plate) were collected 48 h after transfection, washed, and resuspended in 100 μL of PBS buffer. FRET efficiency was measured in a 100-μL quartz glass cuvette using a spectrofluorimeter (LS55, PerkinElmer, Waltham, MA, USA). The fluorescence intensities of mCerulean and FRET at 475 and 530 nm after excitation with a 433-nm light were measured. The ratio between the fluorescence intensity at 530 and 475 nm indicates FRET efficiency. When testing the response of the FRET-HIV sensor to the addition of HIV-1 protease inhibitors, the transfected cells were grown in a medium containing inhibitors (1 μM, final concentration).

### 2.6. Ratiometric Flow Cytometry

For ratiometric flow cytometry, we used a CyFlow Space cytometer (Partec, Münster, Germany) equipped with split optics for 405- and 488-nm light path lines. For excitation of the FRET-HIV sensor and controls, the excitation sources used were a violet diode laser that emits a 100-mW light at 405 nm (donor excitation) and a blue solid-state laser that emits a 50-mW light at 488 nm (acceptor excitation). The optical configuration is shown in Figure 1C. Briefly, after excitation with a 488-nm laser light, fluorescence of the acceptor was detected using a standard 536/40-nm bandwidth bandpass filter (the acceptor channel). To collect the fluorescence of the donor and FRET after excitation with a 405-nm laser light, a 480-nm dichroic mirror and a 450/20-nm bandwidth bandpass filter (the donor channel) and a 520/20-nm bandwidth bandpass filter (the FRET channel) were used. The FRET signal and the mCitrine signal were detected using separate distinct detectors. A side scatter signal was used as the trigger signal. Gated cells were analyzed at low rate settings of approximately 200 cells s^−1^, and at least 20,000 cells were analyzed. Data were analyzed using the FlowJo software (Tree Star, Ashland, OR, USA).

### 2.7. Confocal Microscopy and Sensitized Emission FRET Technique

A Leica TCS SP5 laser scanning microscope mounted on a Leica DMI 6000 CS inverted microscope (Leica Microsystems, Wetzlar, Germany) was used for imaging. The inverted microscope was equipped with an HCX PL APO 63× (NA 1.4) oil immersion objective. The optical configuration is shown in Figure 1B. For sequential excitation, a 50-mW, 405-nm diode laser and a 514-nm line of a 25-mW argon laser were used. Laser power levels of 15% and 2% were adopted for the diode laser and argon laser, respectively.

For FRET experiments, transfections were performed as described above, and the expression levels of the FRET-HIV sensor and controls were adjusted to similar levels by fluorescent intensities. For analysis of FRET with the mCerulean and mCitrine pair, we used the following settings. For the donor and FRET, an emitted fluorescence was detected at 465 to 495 nm and at 535 to 555 nm after excitation with a 405-nm diode laser. For the acceptor, an emitted fluorescence was detected at 535 to 555 nm after excitation with a 488-nm argon laser. The detector gain adjusted in different channels was equal for the FRET and the acceptor channel as required for the sensitized emission FRET method [17]. The image resolution was 512 × 512, the imaging speed was 400 Hz, and 4× magnification was used to obtain pixel dimensions of 120 nm × 120 nm.

The sensitized emission module of the Leica software and PixFRET plug-in (ImageJ, open source, National Institute of Health, Bethesda, MD, USA) was used for the sensitized emission FRET assay. Cells expressing the donor and the acceptor alone were used for calculating the donor and the acceptor spectral bleed-through in the FRET setting. FRET measured in co-expressing cells was corrected for the spectral bleed-through and normalized for expression levels:

(1) $${\text{linNFRET}}_{i}=\frac{{\text{FRET}}_{i}-D_{i}\times (c_{D}\times D_{i}+d_{D})-A_{i}\times b_{A}}{D_{i}}$$

where FRET, *D*, and *A* correspond to the fluorescence intensity of FRET, the donor (mCerulean), and the acceptor (mCitrine) channels, respectively; *b_A_*, *c_D_*, and *d_D_* are constants determined by the fitting of the spectral bleed-through ratio [17].

## 3. Results and Discussion

**Figure 2.**
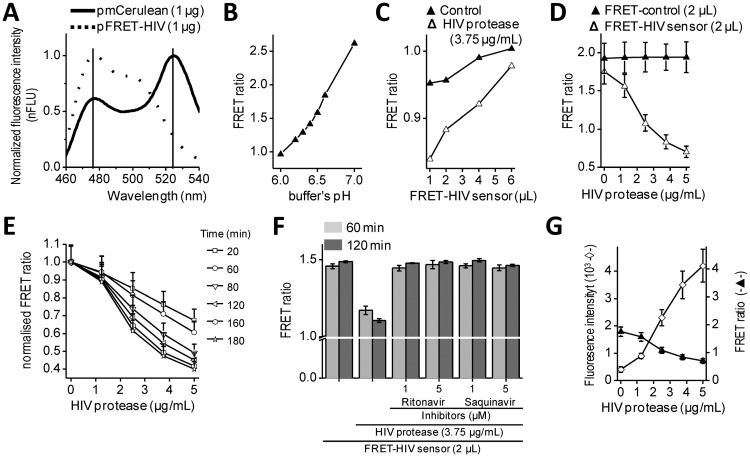
*In vitro* characterization of the FRET-HIV sensor. (**A**) Comparison of emission spectra of HEK293 cells transfected with plasmid coding for the FRET-HIV protease sensor (full line), the mCerulean fluorescent protein (dashed line) excited with a 433-nm light. (**B**) The FRET-HIV sensor is sensitive to pH. The FRET ratios (F_FRET_/F_donor_) of the FRET-HIV sensor in buffers with a pH from 6.0 to 7.0 were determined to optimize the reaction buffer for the *in vitro* HIV protease activity assay. (**C**) Low concentration of the FRET-HIV sensor with respect to HIV protease increased sensitivity of the *in vitro* assay. An optimal ratio between the recombinant HIV protease and the FRET-HIV sensor was determined by varying the concentration of the FRET-HIV sensor at a constant concentration of recombinant HIV protease in the reaction mix. (**D**) A minimal, but still sufficient, concentration of recombinant HIV protease in reaction mix was established by reducing the HIV protease concentration at constant concentration of the FRET-HIV sensor. (**E**) Incubation for 2 h was optimal for the *in vitro* assay. The FRET ratio of the FRET-HIV sensor in the reaction mixtures containing different concentrations of HIV protease were followed for 3 h. (**F**) The *in vitro* assay that we developed was sensitive enough for inhibitor screening. Inhibitors (at a 1-μM final concentration) added to the reaction mixture inhibited the HIV protease, which was detected through the FRET ratio. And (**G**) *In vitro* assay using the FRET-HIV sensor showed similar characteristics as the assay using the HIV Protease Substrate 1. To realistically evaluate the sensitivity of the FRET-HIV sensor for the *in vitro* assays, we conducted *in vitro* experiments using commercially available HIV Protease Substrate 1, which, after being cut with HIV protease, emits a light of 490 nm after excited with a 340-nm light. All *in vitro* experiments were performed in triplicate.

Intramolecular FRET biosensors are often used to examine protease activity, especially in live cell imaging [18]. In this study, we designed a FRET sensor for the detection of HIV protease activity based on a FRET protein pair (Figure 1A). mCerulean was used as donor protein and was linked to the mCitrine, acceptor protein via polypeptide linker that contained the p17/p24 HIV protease cleavage site of SQVSQNY↓PIVQNLQ [12], which is identical to the one in the commercial HIV Protease Substrate 1 (Sigma). In previously described studies, the peptide linker p2/p7 was used in the BRET sensor and in both FRET AcGFP-mCherry sensors [9–11]. Figure 2A shows emission spectra for HEK293 cells transfected with plasmids coding for the mCerulean or FRET-HIV sensor, both excited with a 433-nm light. From the emission spectrum of the FRET-HIV sensor and mCerulean, the contribution of the mCitrine acceptor was clearly visible. When the FRET-HIV protease-sensitive sensor was excited with a 433-nm light, energy was transferred from mCerulean to mCitrine, and an emission peak shifted from mCerulean (475 nm) to mCitrine (529 nm).

### 3.1. In Vitro *Characterization of the FRET-HIV Sensor*

Initially, the usability of the FRET-HIV sensor for *in vitro* measuring of the HIV protease activity was evaluated. Previously described buffers for *in vitro* HIV protease assays were adjusted to low pH values (from 4.7, as in the HIV Protease Substrate 1 assay, to 6.2) [19], which was not suitable for our sensor. The fluorescence of mCitrine (pK_a_ 5.7) and mCerulean (pK_a_ 4.7) is pH dependent and decreases with a low pH [20]; therefore, we screened for the optimal pH of the reaction buffer to obtain an improved FRET ratio of the FRET-HIV sensor (Figure 2B). The buffer at pH 6.5 was selected for further use in all *in vitro* experiments.

Next, an optimal ratio of the HIV protease to the FRET-HIV sensor was evaluated (Figure 2C). Different concentrations of the FRET sensor were added to FRET assay containing a fixed concentration of recombinant HIV protease. We showed that the lower the sensor's concentration, the higher the FRET ratio difference between the cut and uncut FRET-HIV sensors (Figure 2C). We determined that the FRET sensor at a final concentration of 0.2 mg/mL resulted in a sufficient decrease in the FRET ratio in the presence of the HIV protease but still produced a detectable level of the fluorescence signal. An optimal concentration of the recombinant HIV protease needed for efficient degradation of the FRET-HIV sensor was also determined (Figure 2D). To achieve a high FRET ratio dynamic range between the cut and uncut sensor, we analyzed the effect of increasing concentrations of the HIV protease and determined that the concentration of 3.75 μg/mL HIV protease was sufficient to produce a significant decrease in the FRET signal.

The incubation time needed to elicit a high difference in the FRET ratio between the cut and uncut FRET-HIV sensor was evaluated. We determined that a 120-minute incubation period was sufficient and necessary to obtain a good dynamic range between the cut and uncut sensors (Figure 2E). After 120 min of incubation, the difference between the control and HIV protease sample remained almost unchanged; thus, in further experiments, all reactions were measured after 120 min of incubation.

*In vitro* HIV protease assays are usually used in large-scale screening for potential inhibitors. Our *in vitro* assay was therefore tested for such application by the addition of HIV protease inhibitors (Figure 2F). The inhibitors ritonavir and saquinavir successfully inhibited cleavage of FRET sensor even at a relatively low final concentration of 1 μM.

To compare the FRET sensor-based assay to the commercially available one, we performed a synthetic HIV Protease Substrate 1 assay under similar reaction conditions as optimized for the FRET-HIV sensor in buffer recommended by the manufacturer. We observed an 11-fold increase in fluorescence intensity when 5.0 μg/mL of recombinant HIV protease was added to the reaction (Figure 2G). Our FRET-HIV protease sensor showed similar *in vitro* qualities to the commercial synthetic HIV Protease Substrate 1.

The dynamic range of the FRET-HIV protease sensor in the *in vitro* experiments was approximately 2.5; e.g., the calculated FRET ratio for the intact FRET-HIV sensor was 1.8 and in the presence of the HIV protease was 0.7 (Figure 2D). A similar dynamic range was determined for the FRET AcGFP-mCherry sensors [10,11], which is still less than the dynamic range of BRET [9] and the *in vitro* commercial synthetic HIV Protease Substrate 1 (Figure 2G). We have found that even though the synthetic substrates are probably the best choice for *in vitro* screening for potential HIV protease inhibitors, the genetic sensors are superior over the synthetic substrates in *in vivo* assays because the former is produced and applied directly in the cellular environment under analysis and no extra treatment of cells is required.

### 3.2. In Situ *Population Based an Analysis of HIV Protease Activity Using Fluorescence Spectroscopy*

**Figure 3.**
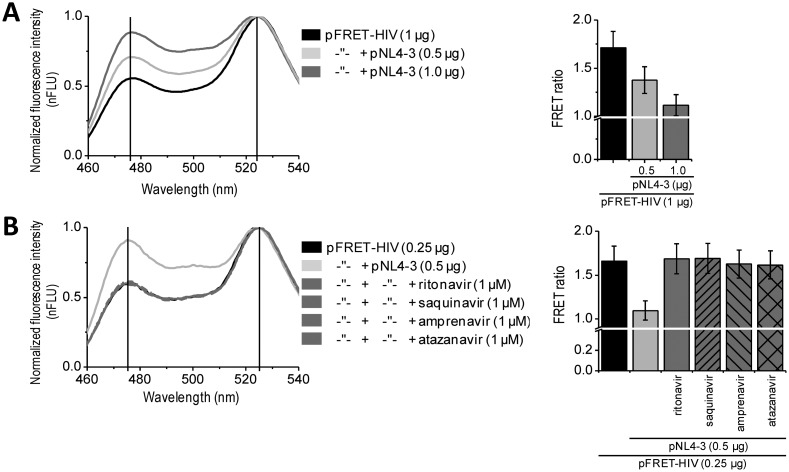
The FRET-HIV sensor was used to detect the HIV protease activity in living cells using fluorescence spectroscopy. (**A**) An emission spectra and calculated FRET ratio (F_FRET_/F_donor_) showed a concentration-dependent response of the FRET-HIV sensor to the intracellular presence of the HIV protease. The FRET-HIV sensor was co-expressed in HEK293 cells with different concentrations of HIV protease (pNL4-3). And (**B**) The addition of a 1-μM HIV protease inhibitor to the growth medium completely inhibited the cleavage of the FRET-HIV sensor in living cells, thus showing the ability of the novel FRET-HIV sensor to be used in large-scale *in situ* screenings for potential HIV protease inhibitors.

*In vitro* assays enable massive screening for potential HIV protease inhibitors. For some applications, such as biocompatibility, membrane permeability, and final screenings, an *in situ* assay would be the method of choice. Such approach better mimics the real-life conditions of a viral infection. We performed *in situ* experiments on HEK293 cells expressing the FRET-HIV sensor transfected with a plasmid pNL4-3.HSA.R^−^.E^−^ (pNL4-3) containing the pseudoviral HIV-1 genome expressing HIV protease. Our *in situ* assay detected expression of the HIV protease. The FRET signal dropped by 25% or 35% when the cells were cotransfected with the FRET sensor and 0.5 or 1.0 μg of the pNL4-3, respectively (Figure 3A). Moreover, the addition of different inhibitors (e.g., ritonavir, saquinavir, amprenavir, and atazanavir) inhibited the FRET ratio decrease caused by the HIV protease (Figure 3B). Taken together, the developed assay could be used for *in situ* screening of the inhibitors of HIV protease.

### 3.3. High-Throughput, Single-Cell FRET Analysis of the FRET-HIV Sensor with Flow Cytometry

Fluorescence spectroscopy is a simple technique for analyzing many samples at the same time. It rapidly records the average FRET ratio of a population; however, it does not provide sufficient single-cell information. A good alternative to spectroscopy is flow cytometry, which combines the rapid online analysis and acquisition of multiparameter results at the single-cell level for each cell in a population. Previously, others have described a flow cytometric optical configuration to visualize cyan-yellow fluorescent protein (CFP-YFP) FRET and its application in monitoring a caspase activity in living cells when a caspase-cleavage site is inserted between CFP and YFP [18]. With this approach, we proposed that HIV protease activity could be measured fast and on the single-cell level using the FRET-HIV sensor.

**Figure 4.**
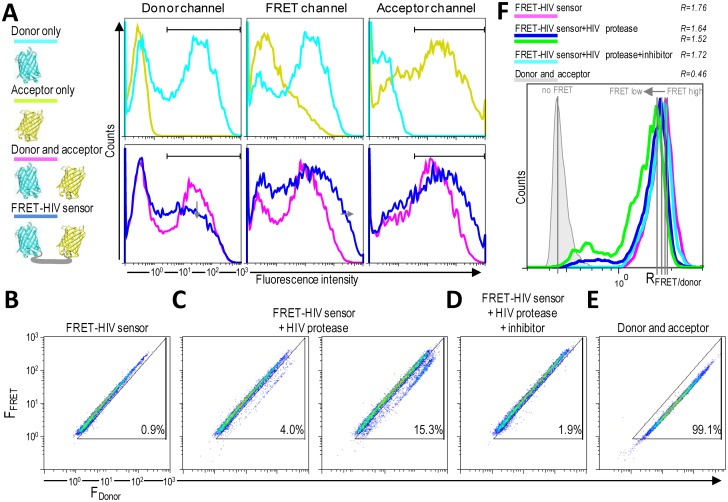
Ratiometric flow cytometry, in combination with the FRET-HIV sensor, was used for single-cell analysis of the HIV protease activity. (**A**) Pulse height emission signals of the donor, FRET, and acceptor of the HEK293T cells transfected with plasmids expressing the FRET-HIV sensor and controls after excitation with 405- and 488-nm lights. The arrows indicate the fluorescence intensity shifts forced by FRET. All profiles were gated from the region in the 2D plot of the forward- *versus* side-scatter (data not shown). The gated populations of cells (indicated by the straight line) represent cells that were further analyzed for FRET efficiency. 2D plots of the fluorescence intensities of the F_FRET_*versus* the F_donor_ of cells transfected with plasmids expressing (**B**) the FRET-HIV sensor, (**C**) the FRET-HIV sensor with the HIV protease without or (**D**) with the protease inhibitor saquinavir (5 μM), and (**E**) the mCerulean and mCitrine expressed separately. Percentage of cells with reduced FRET is indicated. And (**F**) Histogram of the fluorescence intensity (F_FRET_/F_donor_) ratios. Note: The peak of the ratio distribution differed for different type of cells. Depicted flow cytometry plots are representative results of three independent measurements.

HIV protease activity can be monitored by analyzing cell populations with diminished FRET. When FRET occurs, energy is transferred from the excited mCerulean to mCitrine. The energy loss from mCerulean in the FRET-positive cells should be visible as reduced mCerulean emission and enhanced emission in the FRET channel (Figure 4A). A donor-only control (cells expressing mCerulean) showed some spectral bleed-through to the FRET channel. On the other hand, no spectral bleed-through to the donor and FRET channels was detected for an acceptor-only control (cells expressing mCitrine) (Figure 4A).

As shown in Figure 4B–D, two distinct FRET signals in the 405-nm laser pathway were spatially separated. HEK293T cells transfected with only the FRET-HIV sensor exhibited a FRET-high-signal population with more than 99% of the cells showing a similar fluorescence intensity ratio between the FRET channel and the donor channel (*R* = 1.76) (Figure 4C,F). HEK293T cells (more than 99%) cotransfected with plasmids expressing mCerulean and mCitrine (non-FRET control) displayed a FRET-low-signal (Figure 4E). The spectral bleed-through of mCerulean was detected in the FRET channel (Figure 4A). The intensity of the spectral bleed-through was less intense; thus, on a 2D dot blot, the signal was at a different position and the FRET ratio was 0.46 (Figure 4F).

When the cells were cotransfected with the FRET-HIV sensor and pNL4-3, a subpopulation of cells with a FRET-low-signal was detected (3% and 15.3% for 0.8- and 1.5-μg transfected pNL4-3, respectively) (Figure 4C). A calculated FRET ratio peak was 1.64 and 1.52 for 0.8 and 1.5 μg of transfected pNL4-3, respectively (Figure 4F). The HIV protease–induced FRET-low-signal population was abolished when the HIV protease inhibitor was added (Figure 4D), generating a FRET ratio peak of 1.72 (Figure 4F).

### 3.4. In Situ *Characterization of the FRET-HIV Protease Sensor Using Microscopy*

To obtain spatial distribution and a detailed mapping of the FRET in a single cell, the FRET-HIV protein sensor in combination with confocal microscopy was used. We utilized an application wizard from the Leica software and the PixFRET plug-in (ImageJ) [17] to monitor FRET-sensitized emission in HEK293T cells expressing the FRET-HIV sensor.

**Figure 5.**
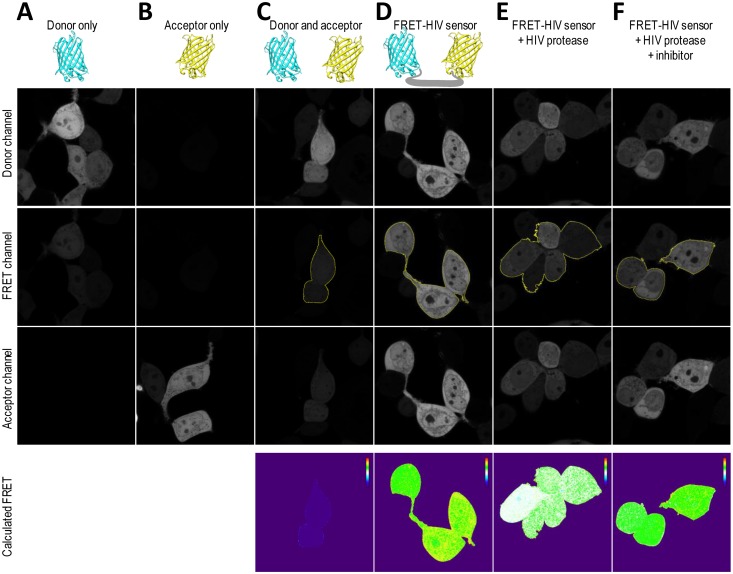
Sensitized emission FRET analysis was used for imaging the spatial expression of the HIV protease in live cells with the FRET-HIV sensor. Images of cells expressing (**A**) mCerulean alone, (**B**) mCitrine alone, or (**C**) mCerulean and mCitrine together, (**D**) and the FRET-HIV protease sensor without or (**E** and **F**) with the transgene HIV protease are shown. Images were taken in the donor [ex. 405, em. 465 to 495 nm], acceptor (ex. 514 nm, em. 535 to 555 nm), and FRET (ex. 405 nm, em. 535 to 555 nm) settings. For the indicated cells, linNFRET images were generated by pixel-by-pixel calculation using the PixFret plug-in (ImageJ) and were pseudo-colorized (violet stands for 0 and red stands for 0.4). Depicted microscopy images are representative results from three independent experiments.

To correct for spectral bleed-through artifacts that could compromise accurate FRET efficiency readings, we acquired images of donor-only mCerulean-expressing cells (Figure 5A) and acceptor-only mCitrine-expressing cells (Figure 5B). All images were acquired in the donor, FRET, and acceptor channels. As seen from the images (Figure 5A), the mCerulean signal bled through to the FRET channel. The fluorescence of mCitrine, on the other hand, showed no bleed-through to the FRET channel (Figure 5B). The cells expressing both mCerulean and mCitrine were used as no-FRET controls (Figure 5C). As predicted, the calculated linNFRET using Equation (1) showed that no energy was transferred between the individual mCerulean and mCitrine. The linNFRET values were presented pixel-by-pixel as a pseudo-colorized image.

We confirmed that the cells expressing the FRET-HIV sensor displayed FRET (Figure 5D). We next asked whether we could trigger a decrease in the FRET of the FRET-HIV sensor cells by cotransfecting the cells with a pNL4-3 plasmid–expressing HIV protease that would be visible through linNFRET calculations (Figure 5E). We already showed that saquinavir inhibited the HIV protease (Figures 2, 3 and 4). We therefore examined FRET change in the cells expressing the sensor and protease in response to saquinavir. We found that the inhibitor efficiently inhibited the HIV protease, which can also be seen in the pseudo-colorized image of linNFRET (Figure 5F).

The sensitized emission FRET technique is probably the most commonly used form of FRET microscopy. It is fast technique that is applicable to live cell measurements. For mCerulean-mCitrine FRET pair is known that the generated FRET signal is weak compared with the spectral bleed-through contribution of mCerulean to the FRET channel; therefore, to properly calculate the FRET ratio images, control samples and images are needed. The AcGFP1/mCherry-mCherry triple combination [11] was analyzed with the fluorescence lifetime microscopy (FLIM)–FRET technique, which is very sensitive and relatively independent of the acceptor and donor concentrations. However, the data acquisition for FLIM is slow, which makes it difficult to apply to living samples [14].

A direct comparison of the AcGFP/mCherry (p2/p7 peptide) sensor [10,11] to our FRET-HIV sensor is not feasible, because in addition to different fluorophore combinations also different techniques are used for analysis of FRET changes. There are some advantages in selecting the mCerulean/mCitrine over the AcGFP/mCherry FRET pair. The mCerulean/mCitrine is most widely used FRET pair; therefore, background literatures are available. In the recent years, mCherry became frequently applied as FRET pair; although it has a disadvantage of a very low quantum yield 0.22 over 0.76 for mCitrine. To overcome the weakness additional mCherry protein was added to the original AcGFP/mCherry pair [11]. Förster radius of the mCerulean/mCitrine pair is 5.4 nm and a dynamic range is between 2.7 and 8.1 nm. For ECFP/mCherry, which is closest mach to AcGFP/mCherry, the Förster radius is 3.5 nm and the dynamic range is between 1.8 and 5.3 nm [21], which might be applied also for AcGFP/mCherry; however it has not been determined, yet. FRET efficiency depends on several parameters to each pair of fluorescent proteins and is directly related to the Förster radius. As the overlap of donor emission and acceptor excitation, the quantum yield of donor, and the acceptor extinction coefficient values increase, so does the Förster radius, which in turn produces larger FRET efficiency. Therefore, it is expected that FRET efficiency of mCerulean/mCitrine pair is higher than of the ECFP/mCherry and also higher than the AcGFP/mCherry, if AcGFP possesses similar absorbance/emission characteristics as ECFP.

When referring to spatial and temporal imaging, we should emphasize that protein based probes introduced in cells as gene fragments on appropriate vectors are fundamentally better than synthetic probes. When fused to a targeting peptide or protein, the genetically encoded FRET-HIV protease-sensitive sensor can be spatially distributed within a cell, enabling protease activity monitoring within subcellular structures that could not be easily performed using synthetic substrates.

## 4. Conclusions

The HIV protease activity was previously measured using a wide range of monitoring techniques, from *in vitro* assays using fluorescent synthetic HIV protease substrates [8] to the use of fluorescent and luminescent protein-based sensors [9–11]. We have found that the use of protein based FRET sensors considerably simplifies measurement, especially when the sensors are expressed in stably transfected cells. *In vitro* characterization of the FRET-HIV sensor that we developed demonstrated sensitivity of the sensor to the HIV protease. Moreover, we have shown that the method using the HIV-FRET sensor is sensitive enough for protease inhibitor screening. The FRET-HIV sensor was tested for the detection of intracellular HIV protease activity in *in situ* assays using fluorescence spectroscopy. This method investigates entire populations and provides only an average measurement of studied parameters. Two additional techniques were developed for more in-depth analysis of HIV protease activity and inhibitor screening. The single-cell analysis by fluorescence microscopy offers spatial and temporal insights into population variability, which we also demonstrated for our FRET-HIV sensor. The ratiometric flow cytometry combines high-throughput analysis at the single-cell level with visualization of FRET distribution within cells populations. The combination of the genetically encoded FRET-HIV sensor and flow cytometry is an effective approach for efficiently analyzing HIV protease activity and inhibitors. Moreover, the ratiometric flow cytometry goes beyond microscopy for high-throughput analysis and screening; it also ensures the fast and automatic postprocessing of obtained data. The disadvantage of the flow cytometry is that it does not allow for the monitoring of particular individual cells; therefore, it can serve as a complementary or alternative solution for microscopy techniques.
